# Needs of amyloidosis patients and their care providers: design & first results of the  _A_MY-NEED_S_ research and care program

**DOI:** 10.1186/s13023-024-03052-w

**Published:** 2024-02-10

**Authors:** Sandra Michaela Ihne-Schubert, Teresa Radovic, Saskia Fries, Stefan Frantz, Hermann Einsele, Stefan Störk, Silke Neuderth

**Affiliations:** 1 Interdisciplinary Amyloidosis Center of Northern Bavaria, University Hospital of Würzburg, Würzburg, Germany; 2 Department of Internal Medicine II, Hematology, University Hospital of Würzburg, Würzburg, Germany; 3 Comprehensive Heart Failure Center (CHFC), University and University Hospital of Würzburg, Würzburg, Germany; 4 Technical University of Applied Sciences Würzburg-Schweinfurt, Würzburg, Germany; 5 Department of Internal Medicine I, Cardiology, University Hospital of Würzburg, Würzburg, Germany; 6 Department of Internal Medicine IV, University Hospital of Gießen and Marburg, Gießen, Germany; 7 Centre for Innovation Research, Lund University, Lund, Sweden

**Keywords:** amyloidosis, needs, amyloidosis-specific care concept, _A_MY-NEED_S_, AmyKoS

## Abstract

**Background:**

Amyloidosis represents a rare yet heterogeneous multi-system disorder associated with a grave prognosis and an enormous psycho-emotional strain on patients, relatives, and caregivers. We here present the overall study design and first results of _A_MY-NEED_S_, a research program aiming to systematically assess the needs of patients suffering from amyloidosis, their relatives and health care professionals (HCPs), and develop an amyloidosis-specific care approach.

**Methods:**

_A_MY-NEED_S_ uses a mixed-methods approach including focus groups (step 1), a questionnaire-based broad evaluation within the local amyloidosis patient collective (step 2), and the development of a needs-adapted care concept (step 3).

**Results:**

Seven patients, six relatives and five HCPs participated in the focus groups (step 1). At the time of diagnosis, patients expressed the need of a smooth diagnostic process, possibly enhanced through improved awareness and better education of local HCPs. There was a strong wish to receive well-founded information and comprehensive support including companionship during medical visits, experience the feeling of being understood, find trust in that “everything possible” is being done, and have effortless access to centre staff. In the course of the disease, patients favoured that the specialized centre should manage treatment coordination, monitoring and psychosocial support. The interface between centre and local HCPs was regarded of particular importance, requiring further investigation into its optimal design.

**Conclusions:**

Patients with amyloidosis express particular needs that should appropriately be considered in specifically tailored care concepts.

**Supplementary Information:**

The online version contains supplementary material available at 10.1186/s13023-024-03052-w.

## Introduction & background

Systemic amyloidosis represents a rare, multi-system disorder resulting from deposition of misfolded proteins in the tissue and consecutive organ dysfunction [1]. The diagnosis of amyloidosis is frequently delayed, because the clinical phenotype is very diverse and lacks early specific signs and symptoms [1–4]. The complexity of this multi-system disease requires interdisciplinary centre-based structures for appropriate diagnostic and therapeutic measures, and facilitation of the interaction of all affected persons, including relatives as well as local and centre-associated health care professionals (HCPs). The journey to diagnosis is burdened with frustrations and disappointments for affected patients associated with a high degree of psycho-emotional stress before, during, and after diagnosis [3].

Previous studies mainly focused on quality of life [5], and patients with systemic light chain (AL) and hereditary transthyretin (ATTR) amyloidosis [5–7]. Subtype-spanning studies also considering treatment requiring ICD-10-coded psychological disorders as well as the type and the extent of treatment-relevant needs of patients and their relatives are lacking. More detailed knowledge of the needs concurring along the diagnostic and therapeutic “journey” of affected individuals, their relatives and carers is required to facilitate needs-adopted care programs that improve adherence and reduce distress.

The _A_MY-NEED_S_ research and care program aims to systematically assess the needs among affected patients with amyloidosis, their relatives and HCPs, and to develop a tailored care concept. Here, we present the rationale, design, and first results of the _A_MY-NEED_S_ program.

## Patients & methods

### *Overall design of *_*A*_*MY-NEED*_s_*& aims*

**Fig. 1:**
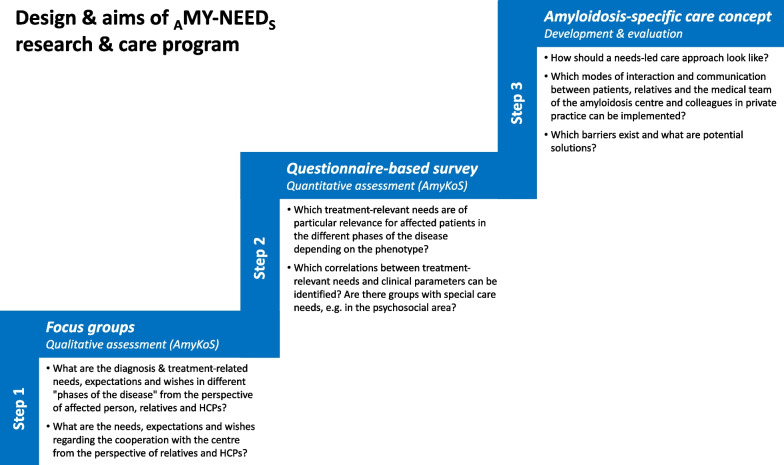
Design and aims of the _A_MY-NEED_S_ research and care program

_A_MY-NEED_S_ was designed as substudy of the local amyloidosis prospective cohort study “AmyKoS”. AmyKoS represents a non-interventional single-centre study at the Interdisciplinary Amyloidosis Center of Northern Bavaria founded in 2018, which includes all patients with suspected or proven amyloidosis who undergo diagnostics and/or treatment at the centre. It aims to systematically collect clinical data as well as biomaterials. Patients are recruited in the outpatient department or during hospitalization by the treating physicians. The _A_MY-NEED_S_ research and care program employs a multi-step mixed-methods approach comprising three consecutive phases (Fig. 1).

#### Step 1: Focus groups to identify possible needs:

Affected patients with amyloidosis and their relatives as well as HCPs were asked about patients’ or their possible treatment-relevant needs, expectations and wishes during the different "disease phases" (a) path to diagnosis, (b) at diagnosis and (c) during the further course of the disease. Three focus groups were planned over 90–120 min with 8–10 participants each by an experienced moderator without medical knowledge about the study participants. A predefined inter-group discussion guide with the following main questions was used:

1. What is particularly important to you in the treatment and care at the amyloidosis centre?
2. Are there special needs in different phases of the disease/treatment?
3. How do you assess the need for support from different disciplines?
4. Looking back, what would have helped you during the course of treatment?
5. How do you experience the cooperation with general practitioners and specialists?
6. What additional support would be useful?

The moderator was assisted by a medical representative of the amyloidosis centre and by an experienced psychologist.

#### Step 2: Quantitative survey on treatment-relevant needs:

Diagnosis and treatment-relevant needs identified during step 1 will be used to design a questionnaire, which on the one hand will serve to quantify patients’ needs and on the other hand will help to identify groups of patients with special needs through correlation with clinical data. Additional aspects of interest are the characterization of living situation, e.g. degree of support such as care level, home care, private expenses due to the health-related restrictions as a result of amyloidosis, e.g. taxi rides, shopping and household help, orthopaedic aids, need for psychological support and potentially available resources in this regard.

#### Step 3: Development and evaluation of an amyloidosis-specific care concept:

Based on the results of the steps 1 and 2 a needs-adopted amyloidosis care concept with a clearly defined evaluation plan will be developed and implemented.

### Study population & setting

For steps 1 and 2, patients were recruited from the still ongoing prospective cohort study in patients with suspected and proven amyloidosis (AmyKoS) conducted at the Interdisciplinary Amyloidosis Centre North Bavaria of the University Hospital of Würzburg. Written informed consent was obtained from all participants prior to any investigation. Patients for step 1 were approached for participation by the local coordinating physician of the amyloidosis centre according to the inclusion/exclusion criteria (Table 1) and paying attention to contrasting the personal characteristics (e.g. age, gender) as well as the disease characteristics (e.g. subtype, pattern and severity of organ involvement). This was to adequately mirror the heterogeneity of the disease also in focus groups. The relatives' focus group was recruited partly from the relatives of the patients interviewed, partly independently. Physicians were selected amongst referring physicians and clinical cooperation partners of the involved departments within the University Hospital based on the distance to the centre, specialisation, and work environment. A deliberately heterogeneous sampling was employed to represent a broad spectrum of views [8], allowing for different combinations of characteristics, in particular experience with amyloidosis and type of specialisations.

**Table 1:** Inclusion and exclusion criteria of _A_MY-NEED_S_ research and care program

		Inclusion criteria	Exclusion criteria
Step 1	Patients	Age at least 18 years	None
		Confirmed amyloidosis according to international criteria	
		Participation in prospective amyloidosis cohort study AmyKoS	
		Written informed consent	
	Relatives	Age at least 18 years	None
		Primary reference person, e.g. relative or close caregiver of a person with proven amyloidosis participating in AmyKoS	
		Written informed consent	
	Health care professionals	Working as a full-time or part-time health care professional (general practitioner, cardiologist, neurologist or haematologist) in private practice, medical facilities or hospitals	None
		Current or past involvement in care of patients with amyloidosis*	
		Written informed consent	
Step 2	All	Participation in the prospective amyloidosis cohort study AmyKoS	None
		Written informed consent	

*The minimum expertise was defined as at least one amyloidosis patient under care

### Data managment and analysis

Within the focus groups (step 1), 7 patients and 6 relatives of patients with amyloidosis as well as 5 HCPs were interviewed. The number of participants for the focus groups corresponded to the methodological standards for group discussions (4–12 participants) according to Schulz et al. [9] and Tausch et al. [8]. General information on the participants was collected by a short questionnaire. The discussions were audio-recorded. After transcription, the audio files were deleted and the transcripts were anonymized. All data analyses of the focus groups were carried out on pseudonimyzed data regarding affected patients (AP1-AP7), relatives (R1-R6) and health care professionals (HCP1-HCP5).

Data analysis was carried out qualitatively and content-analytically by summarizing central aspects of the discussion according to Ruddat et al. [10]. Version 2020 of MAXQDA® was used as the analysis software.

For the analysis, six domains were deductively formed based on the guiding questions:

1. General important aspects of treatment and care at the amyloidosis centre,
2. Special needs in different phases of the disease,
3. Desired support from different groups e.g. HCPs, nurses, patient advocacy groups,
4. Review of possible helpful factors in the course of treatment,
5. Experienced cooperation between the amyloidosis centre, general practitioners (GP) and specialists,
6. Additional support options.

The entire material was coded and corresponding upper and lower categories were created. Subsequently, the material was coded again.

## Results

The focus groups with patients, their relatives and HCPs (step 1) were performed in May and June 2019. Mean duration was 90 min for patients, 76 min for relatives, and 93 min for HCPs, respectively.

The lowest proportion of coded text passages was accounted by AP1 with 18 segments, R4 and R5 with 38 segments each, and HCP4 with 14 segments. Respectively, the highest proportion occurred in AP5 with 79 coded segments, R3 with 80 coded segments, and HCP3 with 97 coded segments. The data material of each focus group comprised 52, 52, and 54 transcript pages; a total of 382, 272, and 274 codes were set, respectively.

### Characterization of focus group participants

#### Patients

Four female and three male amyloidosis patients participated. Their mean age was 67 years (min. 60, max. 73 years). All participants had German citizenship. Three participants did not live directly in Würzburg. Five were married and two were widowed; all had children, three had grandchildren. Four people suffered from ATTR amyloidosis (n = 1 hereditary, n = 3 wild-type), two from systemic AL amyloidosis, one from systemic AA amyloidosis. The time point of diagnosis was on average 2.8 years ago (min. 1.5 years, max. 3 years). The average delay between first symptoms and diagnosis was 1.8 years.

The main symptoms at diagnosis ranged from "no complaints" (n = 2) to shortness of breath, diarrhoea, attacks of weakness and shortness of breath, cough and haematomas. All ATTR amyloidosis patients were under treatment with TTR stabilizer; wild-type ATTR patients were treated off-label (after cost coverage had been obtained by respective health insurance) up to their approval in Germany. One AL amyloidosis patient was in remission (very good partial remission) after first-line treatment with a high-dose chemotherapy with autologous stem cell support; the other AL amyloidosis patient was still under treatment with a combination of antibody and chemotherapy in the 4th line of treatment and had received an orthotopic heart transplant. The patient with AA amyloidosis due to a metastatic inflammatory active solid tumour was treated with tocilizumab. In addition to the amyloidosis centre, the general practitioner (GP; n = 4) and specialists (n = 2) were primary contacts for medical concerns. Two affected patients needed support with everyday activities. Family members (n = 6), friends (n = 3), GP (n = 3) as well as church contact points (n = 1), discussion groups and patient advocacy groups (n = 2) were reported as relevant social resources.

#### Relatives

Four women and two men participated in the focus group. The average age was 63 years (range 43 to 75 years). All participants had German citizenship. Three participants were retired, two worked part-time and one did not give any information.

#### Health care professionals

The average age of the 5 participating HCPs was 42 years (min. 33, max. 52 years), the gender was all male. Two participants were cardiologists, one participant each was GP, nephrologist and haematologist. The median professional experience was 14 years (range 6 to 24 years). Two participants worked in a private practice setting, three as hospital employees. The distance to the centre was 100–150 km for one participant, all others work in the immediate vicinity of the centre (< 50 km). The median number of amyloidosis patients treated over their carreer was four (range 1 to 50 patients). Four of the participants were involved in the treatment of AL and ATTRwt amyloidosis, whereas only two were involved in AA amyloidosis and one each in hereditary ATTR and β_2_-microglobulin-associated amyloidoses.

In the following, we present the needs of the amyloidosis patients that emerged in the three different focus groups from the different points of view, summarised according to the 6 predefined domains mentioned above (Additional file 1: table s1):

### Domain 1

#### Important aspects of diagnosis, treatment and care at the amyloidosis centre

Central aspects for affected individuals can be summarised under the topics “low-threshold access”, “trustful relationship” and “high quality of treatment”. “Low-threshold access” means easy and quick access to help in terms of easy and continuous accessibility of the centre staff, prompt appointments, and quick answering of inquiries. HCPs prefer a low-threshold access, too, but at the same time concerns are expressed about too low-threshold access with regard to overload of the outpatient clinic with extended waiting times and therefore negative impact on the care of urgent patients.

“So that's important, accessible at all times.” (AP1)

Regarding the “relationship” between affected patients and the centre, all groups stress the importance of the continuity and the focus on one permanent medical point of contact/contact person who takes time and "care" e.g. by extensive examinations.

The relationship should be characterized by the feeling of being backed up and accompanied, a sense of being understood and reassured and the trust in that everything possible is being done.

“It's simply the relationship of trust with the attending doctor and also partly with the staff here at the university hospital. That is very, very good.” (R3)

### Domain 2

#### Special needs in different phases of the disease/treatment

Needs differ according to the phase of the disease course. During the journey to diagnosis, the clinical competence of the centre is of particular importance for patients. At diagnosis, there is a special need for psychosocial support and information. In the further disease course and during the treatment, competent treatment, coordination of treatment as well as continuous monitoring by the centre move into the foreground and are perceived as particularly helpful.

#### Needs on the journey to diagnosis

The primary need on the way to diagnosis for patients (and their relatives) is that the diagnosis of the disease is made at all. Patients experience uncertainty on the part of the GPs, as well as a general lack of competence on the part of the doctors contacted. GPs are expected to refer their patients to specialists when necessary and the latter are expected to recognise the disease more quickly. In line with this, the uncertainty of GPs is confirmed from the HCPs’ point of view. The needs are highly dependent on the disciplines. Especially, making of the diagnosis seems to be challenging for HCPs.

“…the eye of the needle is the diagnosis of the disease.” (HCP1)

In this context, the clinical competence of the amyloidosis centre regarding making of the diagnosis is required and appreciated.

#### Needs at diagnosis

At diagnosis, the patients feel lonely and insecure. They raise the issue of inadequate care expressing the need of psychosocial support. The opportunity to talk about the disease with other affected individuals is considered to be important.

“The first thing you hear is that you've been diagnosed and you're alone.” (R1)

Additionally, there is a great lack of information about the disease.

#### During the course of treatment

During the course of treatment, competent treatment counselling by the centre is expected according to HCPs.

“[…] I think that it is also important to have a choice of treatment and counselling, to say, yes, […]” (HCP3)

For patients, the treatability of their disease is of particular importance and they experience the treatment at the centre as "luck". Quality of treatment should follow the current state of research and include necessary resources.

In the case of acute deterioration of the patient, the possibility of inpatient admission for stabilisation should be given according to the GPs. The driving distance to the centre is secondary for patients and their relatives.

According to the HCPs, the relevance of the travel distance to the amyloidosis centre also depends on the general physical condition of the patient. The affected patients prefer coordination of the treatment and continuous monitoring by the centre, as there is uncertainty about the disease and their GPs are often unable to help regarding their questions. However, patients would appreciate better contact between the centre and GPs. Relatives stress the importance of quick feedback from the centre to local HCPs in this context.

### Domain 3

#### Need for support from different treatment groups, self-help and amyloidosis centre: Expectations of medical support

Need for support includes a great unmet need for information (especially well-founded and truthful information), trusting relationships with HCPs and personal manners during interaction with the centre staff are desired.

“Trust is important, information is important and also information about innovations, because there are always changes, maybe new methods or innovations at the moment. Are there studies? It's important to be up to date.” (AP7)

The experience with the nursing support is voted exclusively positively by patients and relatives. Relevant points are for the patients friendliness, helpfulness, prudence, taking time and personal care. Personal care is characterised by the fact that patients are addressed by name and accompanied from ward to ward resulting in the feeling of being seen as a person which seems to be particularly important to the relatives as well.

“You feel that you are addressed by name. That is very important for me, that you are a person and not, in the past, you were a number.” (AP5)

Timely psychological care is important according to relatives and HCPs. Almost all participating patients took advantage of the support offered by the patient advocacy group. The opportunity to talk about the disease and the different experiences beyond the feeling of not being alone were perceived positively by patients and relatives. HCPs showed little knowledge about amyloidosis-specific patient advocacy groups and associated support services.

“I'm sure there is, but I wouldn't know, I couldn't advise my patients now. I have to confess to my shame.” (HCP3)

### Domain 4

#### Review of possible helpful factors/sources of information in the course of treatment

The key factor in the course of treatment represents the provision of reliable and high-quality information. This information is obtained by patients via HCPs, the homepage of the amyloidosis centre, conversations with other patients and the centre staff. Information via free internet search is regarded with scepticism and of low-quality, while information days are evaluated positively by all focus groups. Therefore, relatives and HCPs desire to receive better information about the different courses of the disease at the beginning.

“We did the free light chains and then everything is normal and then I only learned there is wild-type ATTR amyloidosis. I didn't even know that it existed. And that's why he came to us, we didn't diagnose him either.” (HCP3)

Sources of information used in the course of the disease and treatment showed broad variation and were dependent on the level of medical education. For patients and their relatives, the information on the homepage of the centre and provided in personal conversations with the staff of the centre are helpful.

“And what the doctor also told you, what Dr. I. then told me. That was actually also an important source of information.” (AP7)

Therefore, the need of a good internet presence of the amyloidosis centre is highlighted. Relatives and HCPs stress in this context easy access and a well-selected information content. The social environment e.g. family members or friends can be a good source of information too. Information from free internet search does not exactly match the health situation of those affected resulting in anxiety and disconcertion, e.g. regarding life expectancy. HCPs primarily use knowledge databases and colleague networks, but also literature and flyers as sources of their information.

### Domain 5

#### Cooperation between the amyloidosis centre and different groups (relatives, GPs and specialists)

There are mixed opinions on the cooperation between the centre and the GPs. On the one hand, the centre is appreciated for its accessibility, coordination function and competence; on the other hand, relatives in particular emphasised the advantages of a GP model. A better exchange of information between GPs and the centre is considered useful by all  groups. The electronic patient file could be a good support. The targeted inclusion of relatives in the treatment at the amyloidosis centre gives patients security and is highly appreciated.

#### Coordination of the treatment by the amyloidosis centre

The patients are in favor of direct coordination of the treatment by the centre. This is justified by the good accessibility of the coordinating physician and the trust in the centre and its competence. From the perspective of the relatives, opinions are mixed—some of them prefer treatment to be coordinated by the GPs as the central contact person, others by the centre.

“We ALWAYS do this via our family doctor. […] So I think the family doctor model is relatively good, because you have a contact person. Whether the family doctor knows a lot about the disease or not, it doesn't matter now, but in any case everything comes together with him, so that we can then discuss how to proceed.” (A6)

#### Cooperation between GP and centre

Cooperation between the GP and the centre was perceived as partly positive and partly negative by affected patients. Some patients had positive experiences in that they were referred to the right places by GPs and that information was passed on from the GP to the affected person. Other stakeholders and relatives rated the cooperation as negative and asked for better information sharing and cooperation.

“I don't have the impression that the specialist, that the family doctor is informed. I have the impression that the family doctor is groping around blindly […]” (R3)

HCPs would like the amyloidosis centre to organize events and educate local HCPs. In addition, HCPs express a lack of information about the referral process and want clarification on this e.g. by a request form for referral and a flyer.

#### Involvement of relatives

The involvement and information of the relatives is rated positively by the patients, as it is desired by the relatives themselves and gives the affected person a feeling of security.

“But I just feel safer if someone else is listening in.” (AP6)

Relatives appreciate in this context the opportunity to ask questions and to receive some kind of education. However, patients also fear a burden on their relatives.

“I can't say much about it because I only have the daughter and I don't want to burden her, but she knows.” (AP5)

Responsible HCPs consider the involvement of the relatives to be important, as relatives are informed through this. HCPs also share the patients’ opinion regarding the critical aspects of involvement of relatives and therefore argue in favor to a restriction of the range of relatives.

#### Attitude towards the electronic medical record

The electronic medical record was perceived positively by patients and relatives, as it can avoid unnecessary examinations.

### Domain 6

#### Additional support

Affected patients, their relatives and HCPs were asked about their opinion on the following predefined topics: emergency hotline, telemonitoring, psychosocial support and homepage. Additional support through a telephone hotline or telemonitoring is positively assessed by all focus groups. An emergency hotline might result in a relief of doctors and (better) support in crises. Telemonitoring is considered to be rewarding, especially for relatives of patients living in far distance to the centre and for stable patients according to HCPs. Regarding both issues, it is important for HCPs that personal and financial resources are secured. Furthermore, there is a need and desire for psychosocial support: In this context, especially social workers supporting with socio-legal issues are required and rated as important. A brochure containing important legal topics is also desired. HCPs also express their interest in psychosocial case management. Additional support is desired from psychooncologists and palliative care. This could be ensured by expanding the resources of the centre. Regardless of this, affected patients and their relatives expressed the wish for more staff at the centre due to the growing patient collective. For patients, it is important that the existing care is guaranteed.

“Of course, we hope that everything will stay like this, that, let's say, the good care we have here now can be continued. Because somewhere you have to say that the patients are increasing more and more and so far Dr. I. is our, let's say, girl for everything. She is the lifeline for everyone and is, of course, under enormous strain.” (AP7)

## Discussion

The _A_MY-NEED_S_ research program aims to assess needs in patients with amyloidosis, and to develop an amyloidosis-specific and needs-oriented care concept fitting to the German health care system. The used mixed-methods approach has been widely applied for the systematic analysis of needs among various other patient groups including cancer patients [11–14] and formed the basis for the development of tailored health care programs such as heart failure disease management programs [15–18]. Previously, in patients with amyloidosis, McCausland et al. adopted a similar approach to characterize the patients’ journey to diagnosis [3]. However, a subtype-crossing comprehensive analysis of needs including all phases of the disease course and considering the different perspectives of the key players is still lacking. This gap is addressed by the _A_MY-NEED_S_ study. We identified the following dominant needs of patients from the perspectives of patients, their relatives and their HCPs on the journey to and at diagnosis

- the acceleration of diagnosis
- comprehensive support
- exchange of information about the disease

and in the course of treatment

- monitoring and coordination of treatment by a central institution
- close contact between centre and local HCPs, especially active involvement of GPs.

The unmet need of accelerated diagnosis is in line with earlier data showing establishment of diagnosis in light chain (AL) amyloidosis ≥ 1 year after the onset of initial symptoms in 37% and requiring contact with ≥ 5 physicians in 32% of participating AL amyloidosis patients [2] resulting in severe disease burden. Another study demonstrated that 42% of patients with ATTRwt amyloidosis and a cardiac phenotype experienced a delay of > 4 years of diagnosis after first presentation with cardiac symptoms [4]. Of note, patients with cardiac AL amyloidosis and thus an exceptionally poor prognosis receive their diagnosis particularly delayed [3], which stresses the clinical relevance of this need. Possible solutions for these problems proposed by the participants included improved awareness and education of local HCPs (GPs and specialists). This concurs with discussions triggered by now established non-biopsy diagnosis of cardiac wild-type transthyretin (ATTRwt) amyloidosis [19] and treatment options that have been approved [20] or are in the advanced stage of clinical investigation (e.g. NCT04136171, NCT03997383).

Although already published data about cancer patients suggests that unmet needs appear to concern particularly the treatment phase [21], our focus groups emphasized that the disease-specific unacceptably long journey to diagnosis is even more critical than the treatment phase itself. However, these conclusions require confirmation in step 2 of our research agenda. The reported feeling of being left alone and the unmet need for information also fit well to data published by others showing that the primary source of information are centres’ and other organizations’ own material in 39% and 34%, but also patient advocacy groups in 29% [2]. However, as reported for cancer patients [21], a large proportion of patients (40%) received no information material at all [2].

As expected, there was a high level of trust on the side of patients into the abilities of the centre and its provided care. In particular, medical competence and the human approach were repeatedly emphasized.

Patients’ hope for a comprehensive support provided by the centre with the options of easy and continuous accessibility and personal contact was connected with the patients’ wish that treatment and monitoring coordination should be managed by the centre rather than by the individual HCPs. Whereas such desire is understandable, its implementation into routine care still needs to be defined.

Owing to the individual clinical phenotype of amyloid disease, every patient requires an individually assembled team consisting of the GP, local specialists and the multidisciplinary medical team of the centre resulting in a multiplication of contact persons and communication processes. Patients, their relatives and their HCPs expressed disparate needs beyond common wishes (i.e., easy and continuous accessibility, low-threshold access, immediate reactions). These expectations, e.g. target group adapted information and education, are challenging both in terms of time and content and are not addressed in the current health care system so far.

Telemonitoring and remote care concepts including the electronic medical record received a positive evaluation. Their feasibility and clinical benefits have already been demonstrated by examples from the rare disease area [22, 23] and have been applied to common diseases such as heart failure, e.g. HeartNetCare-HF™ program [15–17]. The post-discharge disease management program HerzMobilTirol, which has already been integrated into the Austrian health care system, proved beneficial in terms of mortality risk reduction [24]. Against this background, the integration of these aspects into a comprehensive care concept should be examined.

Limitations of the results presented here result from the small group size (although within normal range of focus groups), the single-centre approach, and a possible selection bias of participants, e.g. through predominant recruitment via the patient advocacy group. Nevertheless, it was possible to map several perspectives within the individual groups as well as through the three groups themselves by internal and external assessment of the needs of those affected. Patients and their relatives as well as professional care givers had the opportunity to participate in a targeted improvement of disease-specific care according to a participatory approach, which represents a novelty in the care of patients with amyloidosis. Finally, we propose a generally applicable approach to analyse the often overlooked and insufficiently investigated psychosocial and patient-centred aspects in rare diseases, using amyloidosis as an example, which may be transferable to other rare diseases and expandable to a multi-centre approach.

## Conclusions

The _A_MY-NEED_S_ research and care program represents the first comprehensive assessment of needs in amyloidosis patients during their disease course with the overall aim to develop a needs-driven, tailored care concept. According to the preliminary results of step 1, centralized care is well-accepted in amyloidosis patients. Unmet needs focus in particular the critical phase of the journey to and making of diagnosis, the further development of the centralised care approach with regard to multidisciplinarity and multidimensionality with the inclusion of paramedical disciplines such as social workers and psychologists. The interaction between the centre and HCPs is a critical interface. Confirmation of results is required by step 2 of our research program.

### Supplementary Information

**Additional file 1. Table 1:** Categories.

## Data Availability

Please contact author for data requests.

## Code availability

MAXDA® version 2020.
