# Supplementary material for: Needs of amyloidosis patients and their care providers: design & first results of the AMY-NEEDS research and care program
Source: Orphanet J Rare Dis. 2024 Feb 10;19:58. doi: 10.1186/s13023-024-03052-w (PMC10859020; doi:10.1186/s13023-024-03052-w)
Supplement: Supplementary file 1 — Additional file 1. Table 1: Categories. [file 13023_2024_3052_MOESM1_ESM.docx]

***ONLINE SUPPLEMENT***

**Table 1** *Categories*

| **Domain** | **Top category** | **Relevant subcategories** | | |
| --- | --- | --- | --- | --- |
|  |  | ***Patients*** | ***Care givers/relatives*** | ***Health care professionals*** |
| 1. ***Important aspects of treatment and care at the amyloidosis centre*** | In contact with the centre | - constant accessibility - timely appointments | - constant accessibility - timely appointments - fast processing of enquiries | - easy accessibility - timely appointments - possibility of short-term connection - low-threshold access, but also concerns about too low-threshold access - Agreement regarding access |
|  |  | *“So that's important, accessible at all times.” (AP1)* | *“Also the availability if anything happens. You write an e-mail and get an answer immediately.” (R1)* | *“Double-edged sword. If access is easy and all patients can come, then under certain circumstances the outpatient department can become full relatively quickly and then perhaps with many cases that could be treated relatively easily on site. They then lose time, wait for the appointment, then the appointment comes, then you are referred back again. In the meantime, other people who might have been seen more urgently might not have been seen. So it is difficult to control this so that the patients who need special care are seen early. Others, who can perhaps be treated on site, are always at a disadvantage.” (HCP3)* |
|  | Support by centre employees | - what is possible is done - well-founded information - personal contact - clear contact person - trusting relationship - feel understood - be calmed | - feel safe - well-founded information - personal contact - clear contact person - trusting relationship | - well-founded information - focused contact person |
|  |  | ***“****That I, that I feel understood.” (AP5)* | ***“****It's simply the relationship of trust with the attending doctor and also, to some extent, with the staff here at the university hospital. That is very, very good.“ (A3)* | ***“****I think that because it is a rare disease, it is important that there is as concentrated a contact person as possible. For the patient in particular, because he or she is also uncertain and does not receive adequate information in many places, which makes him or her even more uncertain. That's why it's important that there is a doctor who they can turn to again and again and who guides them through the whole thing. (P5)“* |
|  | During treatment at the amyloidosis centre | - extensive investigations - taking the time of the doctors - treatment options | - extensive investigations - regular checks | - Ensure the necessary resources - treatment according to current state of the art |
|  |  | *“Yes, I would only say that we have illness, we will always have it, but it can be contained and that is the most important thing for me. I think that is the case with everyone, we have it and they can't make it go away but they can make it more tolerable.” (AP5)* | “*My wife was very satisfied (unv.) with the extent of the examinations, which somehow reassured her. That she was really (unv.) examined and everything was taken into account.” (R3)* | *“For me it would be important that, I'll just start, the latest and possibly experimental treatments in connection with studies are available for my patients. That when I send the patients there, they get something that they don't get here. Newer therapies and perhaps experimental ones, and that more comprehensive care is then possible, especially for patients who now have complex problems or are younger, where we want to achieve more than we do now with older people whom we perhaps treat locally.” (HCP3)* |
| 1. ***Needs in different phases of disease and treatment*** | Journey to diagnosis | - diagnosis of the disease | - diagnosis of the disease | - different needs of the various disciplines - uncertainty of GP |
|  |  | *“Well, the fact that it has been established at all what we have. Nobody knew that before. I've contacted to so many different clinics and always because of weakness and no one has found anything. There were also numerous examinations, but no one ever told me that the heart wall was thick, but no one ever told me what to do and whether anything could be done.” (AP7)* | “*The main problem is the recognition of the disease by other specialists. That is actually the main problem and too much time is lost for that.” (R3)* | *“As a GP, at what point do I have to think about this, if the creatinine values rise as first symptom of patients, or how? Or can I do that myself? Let's assume that I don't have such an (unv.) patient here in the centre every day. How can I rule out that someone has amyloidosis?” (HCP2)* |
|  | At diagnosis | - comprehensive support - exchange about the disease | - exchange about the disease | N/A |
|  |  | “*Yes, the beginning is very simple for me and I think very, very important for many people and also for the relatives, I have to say, because you don't know, you don't know. I mean, when you talk about other diseases nowadays, you have a bit of background, then you know where you can go straight away. You don't, you can't talk to anyone about why it is like that.” (AP5)* | *“We haven't had an interlocutor yet, we've only had the diagnosis and then you inform yourself and you are confronted with it, that was tough.” (R1)* |  |
|  | In case of crises and restrictions | N/A | - rapid feedback - support through psychological care | - Inpatient stay in case of acute deterioration - Support through psychological care - treatment counselling |
|  |  |  | *“That was also the case with us. If the values were somehow in the basement again or if something was wrong, but then it was, yes, we're back to the topic that you get information quickly here or by email and then the callback is also quick. You are then more or less reassured and also receive suggestions for remedial action, which has already worked well.” (R1)* | *“I also think that the choice of therapy is still important, because you can treat it in different ways, you can treat it palliatively, that stops the symptoms a little bit and you can treat it aggressively if you can get rid of it and that it doesn't come back and of course we don't want that, that patients do not benefit from a potentially curative therapy and that other patients do not unnecessarily receive an aggressive, dangerous and possibly also risky therapy and could be spared this, so I think that it is also important to have a choice of therapy and counselling, to say, yes, the patient can be treated palliatively and send him back to you. Can the resident oncologist do it or no, we keep him here, he gets a bone marrow transplant, we make him healthy again, would be good.” (HCP3)* |
|  | under treatment | - close contact between the centre and GPs - monitoring by the centre - coordination of treatment by the centre | N/A | N/A |
|  |  | *“So it is also important that the family doctors are perhaps better involved in the further­ treatment or in the treatment in between. Also with vaccinations, yes, can I have the vaccination, can I not have it done? When can I have it done? That is always a problem for me, for example, can I have, I don't know, tetanus vaccination, can I have any other vaccination, that is always a problem.” (AP7)* |  |  |
|  | Distance to the centre | - short way practical - distance is secondary | - short way practical - distance is secondary | - distance depending on physical condition |
|  |  | *“They also put up with me. We simply said that if I have a better treatment option there, then I'll put up with the fact that I have to drive.” (AP4)* | *“Well, we have a three-hour drive to Heidelberg and that is of course very lucky for us that we only need half an hour to get here.” (R2)* | *“It is probably the case that the further the distances, the younger the patients who come along, because young people with a potentially higher life expectancy are more likely to take on the long journey if they are still physically fit, than if they are 80-year-olds from Hamburg or elsewhere.”*  *(HCP2)* |
| 1. ***Need for support from various groups of health care providers, self-help amyloidosis centre*** | Expectations of medical support | - enlightenment and truth - trusting relationship - specific information on new treatment options | - enlightenment and truth |  |
|  |  | *“and also information about innovations, because there are always changes, maybe new methods or whatever innovations are coming at the moment. Are there studies? That you are a bit up to date. I think it is important to know that something is being done or where something is being done.” (AP7)* | *“Yes, honesty too. As I said, it's not peace, happiness, pancakes and so on, but also honesty, I think that was important to my wife. She must know that.“ (R3)* |  |
|  | Assessment of nursing support in the amyloidosis centre | - Friendliness - helpfulness - prudence - timekeeping - be seen as a person | - helpfulness - be seen as a person | N/A |
|  |  | *“You feel that you are addressed by name. That's very important for me, that you are a person and not, in the past, you were a number.” (AP5)* | *“In the other building, my wife was going up the stairs and climbing stairs causes her problems. She stopped on the landing and puffed for a moment, a nurse passed by at the top, "can I help?" I thought that was kind of great. He had nothing to do with us, he didn't know us. He just went upstairs and said, "Can I help?" Good, wonderful. I thought that was great.“ (R3)* |  |
|  | Assessment of the psychological support | - good service - individual adaptation - timely date | - timely date | N/A |
|  |  | *“Yes, we always discuss this individually, but it always fits.” (AP6)* | *“Yes, because if it's like that, in a normal practice, they have waiting times of half a year. And when you get a diagnosis like that, you don't need the session in six months, you need it now. And that happened relatively quickly, I have to say. So within a few weeks we have an appointment and that also works quite reliably, regularly. They are always in contact, if something is postponed for whatever reason, it works well.” (R6)* |  |
|  | Support through patient advocacy group | - common exchange - different experiences - feeling of not being alone | - common exchange - feeling of not being alone | - lack of knowledge |
|  |  | *“You can exchange ideas with each other and that's good in a way.” (AP3)* | *“No, it's all right, I'm not alone, like this. I'm not alone in feeling so bad, that's what I mean. But that helps, helps a lot.“ (R3)* | *“I'm sure there are, but I wouldn't know, I couldn't advise my patients now. I must say to my shame.“ (AP3)* |
| 1. ***Review: Potentially helpful aspects in the course of treatment/information during the course of treatment*** | Helpful information at the beginning | N/A | - better information about different courses | - better education about different courses of disease |
|  |  |  | *“Yes, and that was just not the case. But that was his statement. And then we went to Heidelberg and then here. But what all that entails now, also with my husband, who is no longer allowed to work and so on, was put on sick leave from one day to the next, pulled out somewhere, so and that was not known at the beginning. I mean, it wouldn't have changed anything, but yes, there would have been the clarification, so that was actually that the doctor wasn't that familiar with it.” (R3)* | *“We did the free light chains and then everything is normal and then I only learned there is wild-type ATTR amyloidosis. I didn't even know that it existed. And that's why he came to us, we didn't diagnose him either. It is a multifaceted clinical picture or syndrome, or rather, it contains various diseases that manifest themselves differently. (HCP3)* |
|  | Assessment of the sources of information used in the course of treatment | - positive experience through social environment - helpful information through the centre’s homepage - helpful information through personal conversation - negative emotion due to information from the internet | - helpful information through an information event - negative emotion due to information from the internet | - use of knowledge databases - literature - colleague network - flyer - information events |
|  |  | *“And what the doctor also told you, what Dr. I. then told me. That was actually also an important source of information.” (AP7)* | *“We did some research on the internet when we found out the diagnosis and it was awful.” (R1)* | *“And when the patient was diagnosed, I of course read up on the internet and new studies, etc. and there was a patient and family day right at the beginning, where there were also various lectures from different disciplines. I was there and it was very informative.“ (HCP2)* |
|  | Organisational aspects | - difficulties with approval of treatment and medicines by health insurance | N/A | N/A |
|  |  | *“That is one of the greatest difficulties for the patient, because then the patient has to fight and I think, I understand, I know that the health insurance companies cannot pay for everything, some of the things are very expensive, but these are university hospitals nonetheless. So I think we need to publish this more in public, so that people don't have to fight such a desperate battle. I think that's because you have enough to do with the diagnosis and then you often don't have the strength to take care of such things. And I think that's actually quite bad if someone is on his own and then does not [or] receive it too late.” (AP7)* |  |  |
| 1. ***Cooperation/ Experienced cooperation between amyloidosis centre, GPs and specialists*** | Coordination of treatment by GP |  | - Central contact |  |
|  |  |  | *“We ALWAYS do this via our family doctor. We ALWAYS have all the findings sent to her, so that everything really comes together in one place, and we always discuss it with her. Because from our point of view, it's not worth it if you send one part to your family doctor and one part to your cardiologist. We have always done it that way. With us, everything comes together at the family doctor's and so the only thing is that the findings, if they came a little earlier, it wouldn't be bad, but this way we can always go to the family doctor, she always reads through it and can always give us the lab results, so she could always clarify them. So I think the family doctor model is relatively good, because you have a contact person. Whether the family doctor knows a lot about the disease or not, it doesn't matter now, but in any case everything comes together with him, so that we can then discuss how to proceed. That's how we have it.“ (R6)* |  |
|  | Coordination of treatment by the Amyloidosis centre | - accessibility of centre – employees - trust in competence | - trust in competence | N/A |
|  |  | *“Yes, directly with the centre. Dr. I. is always available if there's anything wrong. That's good.“ (AP3)* | *“Yes, because the family doctor doesn't really know what he's talking about. He said to me at the time, "Yes, nowadays you can examine everything. In the past, you couldn't determine that at all, and today you just do such lab values", but I don't think he really knows his way around that well either. It's better to be looked after in the centre and get the information there.” (R2)* |  |
|  | Cooperation between GP and centre | - better information transmission - better cooperation | - better information transmission - better cooperation | - organisation of events - education of local doctors - cooperation with pharmaceutical companies - request slips for relief - support through a flyer - education about referral |
|  |  | *“When I go to the GP and say I have a new report, because they don't read it if they don't and they don't know that either. I say I want to know what is written there and whether they understand it better than I do. And then they do that and then it works. But only when I talk to them, not on their own. So when GPs get a report, they should read it automatically.” (AP4)* | *“I don't have the impression that the specialist, that the family doctor is informed. I have the impression that the family doctor is groping around blindly and then asks my wife. "And have you?" "No, I haven't got anything yet, I haven't got anything yet, I haven't got anything yet." For weeks after she's been in, yes. She has no results. So from my point of view, the cooperation or information with the GP doesn't work very well.” (R3)* | *“Just shake up the local doctors or local systems that are here and show that this is here and you have to pay attention to the symptoms and then more will be diagnosed.” (HCP5)* |
|  | Assessment of the involvement of relatives | - sense of security through relatives - fear of burdening relatives - clarification of relatives | - clarification of relatives | - education of relatives - restricting the range of relatives |
|  |  | *“But I just feel safer if someone else is listening in.” (AP6)*  *“I can't say much about it because I only have the daughter and I don't want to burden her, but she knows.” (AP5)* | *“Yes, sometimes yes, but somehow that's, you can ask everything, no matter whether it's the patient himself or (unv.) you can take everyone along for a talk, that was already good.” (R4)* | *“I can only agree with that. This is absolutely the practice that the colleagues have described and also the difficulties that arise when too much information is given, contradictions arise and the need for information is of course there, the family is worried about the relative but there are also conflicts within the family, that is also normal everywhere. They also manifest themselves there and we, as doctors, simply have to hold back a bit and conserve resources.” (P1)* |
|  | Attitude towards the electronic medical record | - positive attitude towards the electronic medical record | - positive attitude towards the electronic medical record | N/A |
|  |  | *“and then I could present them if something happens or something. So I don't have a card, because I still have a CardioMEMS thing inside and then I have certain parts in my shoulder, new parts and so on, and for each one I have a card and I'm supposed to show them all. And on the electronic one everything would be stored on it.” (AP7)* | *“It would have to be well secured, but of course yes. This would also prevent the same examination being carried out five times, in part.” (R3)* |  |
| 1. ***Additional support*** | Emergency hotline | - interest in emergency hotline | - interest in emergency hotline - relieving the burden on doctors - crisis support | - securing human resources |
|  |  | *“I also find that very interesting.” (AP7)* | *“That's right, as they say, we haven't had any crises with us yet, but that's really true. What do I do now when things get really serious? Then I'll be in a pretty bad position, I admit. Yes, yes, that wouldn't be wrong, yes.” (R3)* | *“Yes. Emergency hotline. Who is supposed to sit at the end of the phone line? Then you have to do social service, which is then called at night.” (HCP3)* |
|  | Telemonitoring | - interest in telemonitoring | - interest in telemonitoring | - interest in telemonitoring - securing financial resources |
|  |  | *“I find that quite interesting.” (AP1)* | *“It's certainly not bad. Also for those who have long distances to travel. So, like for us. I mean, I don't know, you can drive four kilometres here and because of, let's say, little things. All other things are certainly not bad for a long distance.” (R5)* | *“Especially for patients who are stable. If someone has a long way to travel, they are not routinely called in every quarter or every six months, but they can then simply pass on their subjective assessment of breathing difficulties, weight and blood pressure values via tele-monitoring. So, what patients can also do themselves, with the support of the family doctor, when it comes to laboratory and such important things. Especially for stable patients, I think.“ (HCP2)* |
|  | Social worker | - desire for social legal support from social workers | N/A | - securing human resources - support with socio-legal issues - interest in case management |
|  |  | *“Or a brochure [explaining] the legal aspects, like a timetable, you can do that, you can't do that, are you entitled to that or aren't you entitled to that, or that you can (unv.). “(AP5)* |  | *“Yes, to everything. They have mobility problems, oedema, things like that, bone pain, they can maybe apply for a severe disability card but they have to manage that first. In addition, they have to apply for assistance, a care level and all these complicated things for which, I think, the doctors have neither the time nor the knowledge to help the patients. When he comes with a form, my heart sinks when I see the forms I have to fill out in my signature folder. Then I think, oh man, I'd better do that later. So it's good to have someone who is employed for that and specialised in that.” (HCP3)* |
|  | Homepage | - good internet presence of the Amyloidosis Centre | - faster accessibility of the homepage | - interest in homepage |
|  |  | *“I find the information from the centre, it helped me. Great internet, that's how it was for me. And they spoke clear words and you were actually more reassured, like with the internet.” (AP2)* | *“That you get to this page as quickly as possible, because there really is a lot of rubbish in there about them.” (R3)* | *“An easily accessible website that is clear and contains the information is certainly always useful.” (HCP3)* |
|  | Fear of staff overload at the amyloidosis centre | - desire for increased staffing - securing the existing supply | - desire for increased staffing | - support from psychooncologist |
|  |  | *“Of course, we hope that everything will stay like this, that, let's say, the good care we have now, that it can be continued.” (AP7)* | *“It was always very short. (unv.) And that is also my fear here in Würzburg, if I may say something negative. So at the moment it's going really well, really true. My only fear is that Dr. I. is only human and that she won't be able to keep it up in the long run the way she is doing it now. So that's really my fear. So, as I said, we were with Dr. H. and they have ten times the amount, or so it seems, and you went in every minute, in out out out out, she also worked excellently with e-mail, sometimes an e-mail came from her at half past eleven at night and for me that is a sign that the woman works around the clock and that is little my fear here that Dr. I. will also reach her limit in the course of the next few years. That's what I wanted to say, that's what I really have to say.” (R6)* | *“Yes, then psycho-oncology. People then live with this uncertainty all the time. Will it come back, etc.? That is also important. And palliative care, yes, there is still too little of that in Germany. The amyloidosis centre can't do all that, it's not possible. It's not feasible. There is a need for a connection or at least information that there are palliative care centres or practices or hospices or something and then, if the therapy is no longer effective, then of course also how to get there. That there is something like that, where it exists. And the patients come from far away, not only in Würzburg and the surrounding area, but you need someone who can look for them, who can find them, because they can't do it themselves. They are sick and weakened and they don't have the courage to somehow get on the phone and call, "Do you have an appointment for me? I am seriously ill. They don't do that.“ (HCP3)* |
